# Echocardiographic Assessment of Mitral Valve Prolapse Prevalence before and after the Year 1999: A Systematic Review

**DOI:** 10.3390/jcm13206160

**Published:** 2024-10-16

**Authors:** Andrea Sonaglioni, Gian Luigi Nicolosi, Antonino Bruno, Michele Lombardo, Paola Muti

**Affiliations:** 1 Division of Cardiology, IRCCS MultiMedica, 20123 Milan, Italy; michele.lombardo@multimedica.it; 2 Division of Cardiology, Policlinico San Giorgio, 33170 Pordenone, Italy; gianluigi.nicolosi@gmail.com; 3 Laboratory of Innate Immunity, IRCCS MultiMedica, 20138 Milan, Italy; antonino.bruno@multimedica.it; 4 Laboratory of Immunology and General Pathology, Department of Biotechnology and Life Sciences, University of Insubria, 21100 Varese, Italy; 5 Department of Biomedical, Surgical and Dental Sciences, University of Milan, 20138 Milan, Italy; pmuti26@gmail.com; 6 IRCCS MultiMedica, 20099 Milan, Italy

**Keywords:** mitral valve prolapse, prevalence, general population, M-mode echocardiography, two-dimensional echocardiography, diagnostic criteria

## Abstract

**Background:** Over the last five decades, a fair number of echocardiographic studies have evaluated the prevalence of mitral valve prolapse (MVP) in various cohorts of individuals, including heterogeneous study populations. The present systematic review has been primarily designed to summarize the main findings of these studies and to estimate the overall MVP prevalence in the general community. **Methods:** All echocardiographic studies assessing the MVP prevalence in various cohorts of individuals, selected from PubMed and EMBASE databases, were included. There was no limitation of time period. The risk of bias was assessed by using the National Institutes of Health (NIH) Quality Assessment Tool for Observational Cohort and Cross-Sectional Studies. **Results:** The full texts of 21 studies with 1354 MVP individuals out of 63,723 participants were analyzed. The overall pooled prevalence of MVP was 4.9% (range of 0.6–21%). When dividing the studies in two groups according to the echocardiographic criteria used for MVP diagnosis (less specific old criteria or more specific new criteria, respectively), the estimated pooled prevalence of MVP was 7.8% (range of 2–21%) for the older studies (performed between 1976 and 1998) and 2.2% (range of 0.6–4.2%) for the more recent ones (conducted between 1999 and 2021). Potential selection bias, hospital- or referral-based series, and the use of less specific echocardiographic criteria for MVP diagnosis have been indicated as the main reasons for the higher MVP prevalence detected by the older studies. MVP was commonly associated with a narrow antero-posterior thoracic diameter, isolated ventricular premature beats and nonspecific ST-T-wave abnormalities on a resting electrocardiogram, mild-to-moderate mitral regurgitation (MR), the reduced probability of obstructive coronary artery disease, and a low frequency of serious complications, such as severe MR, infective endocarditis, heart failure, stroke, and atrial fibrillation. **Conclusions:** MVP has a low prevalence in the general population, regardless of age, gender, and ethnicity, and is associated with a good outcome.

## 1. Introduction

Mitral valve prolapse (MVP) has received great attention throughout the 1970s, 1980s, and 1990s due to its frequent detection on transthoracic echocardiography (TTE) and, most of all, for a number of serious complications ascribed to this valvular disorder, including infective endocarditis (IE), ventricular and/or atrial arrhythmias, stroke, heart failure (HF), and mitral regurgitation (MR), requiring surgery [1,2,3,4].

The echocardiographic studies that evaluated MVP prevalence before the 1999s predominantly analyzed select individuals, hospitalized due to MVP-related complications or “self-referred” because of affected family members and/or with a history of heart murmur or subtle symptoms. Moreover, they used motion-mode (M-mode) or two-dimensional (2D) echocardiographic criteria that were not specific for MVP diagnosis. According to M-mode criteria, MVP is generally defined as late or holosystolic bowing of mitral valve leaflets at least 2 mm below the C-D line [5,6], whereas on 2D-TTE, MVP is diagnosed in the case of systolic motion of one or both mitral leaflets above the mitral annular plane, at least in the apical four-chamber view [7,8]. Such criteria do not take into consideration the three-dimensional shape of the mitral valve apparatus [9,10]. Due to the saddle-like shape of the mitral annulus, leaflets can appear to ascend above the mitral annulus in the apical four-chamber view, without real leaflet displacement above the entire mitral valve in three dimensions.

The frequent echocardiographic diagnosis of MVP could have negative effects on young, otherwise healthy persons, such as anxiety about the possibility of adverse events, ineligibility for insurance or competitive sports, and the need for antibiotic prophylaxis [11].

The studies demonstrating systolic mitral annular nonplanarity [9,10] give input for reconsidering the echocardiographic standards for MVP diagnosis.

Based on the new 2D-echocardiographic criteria, since 1999, MVP has been diagnosed as the systolic billowing of one or both mitral leaflets > 2 mm above the mitral annulus into the left atrium in the parasternal long-axis view [12]. These new echocardiographic criteria have allowed for the minimization of false positive diagnoses [13,14].

The present systematic review has been primarily designed to summarize the main findings of the most relevant echocardiographic studies that assessed MVP prevalence in various cohorts of individuals, including heterogeneous study populations, from the 1970s to today. Possible explanations for the divergences between the studies conducted before and after the year 1999 will be provided as well.

## 2. Materials and Methods

This systematic review was performed according to the Preferred Reporting Items for Systematic Reviews and Meta-analyses (PRISMA) guidelines [15] and was registered in the INPLASY database (registration number INPLASY202480123).

### 2.1. Search Strategy

A comprehensive search of all echocardiographic studies estimating MVP prevalence in various cohorts of individuals, regardless of the time period, was carried out by two independent reviewers (A.S. and M.L.) through August 2024 by using Medline and EMBASE databases. The search strategy included the following terms: “mitral valve prolapse” OR “MVP” AND “prevalence” AND “cardiac function” AND “echocardiography” OR “M-mode echocardiography” OR “two-dimensional (2D) echocardiography” OR “three-dimensional (3D) echocardiography”. The search was limited to full-text articles published in English.

### 2.2. Eligibility Criteria

All echocardiographic studies evaluating MVP prevalence in various cohorts of individuals, regardless of the time period, were included. Conversely, imaging studies conducted on MVP individuals that did not analyze MVP prevalence, non-clinical articles, animal studies, duplicate articles, case reports, conference presentations, reviews, correspondences, editorials, letters without data, and abstracts, were excluded.

### 2.3. Study Selection and Data Extraction

Two reviewers (A.S. and M.L.) screened the databases according to the inclusion criteria and performed data extraction independently. The following information concerning MVP individuals was independently collected by the two reviewers: (1) demographics (age and sex); (2) anthropometrics [waist-to-hip ratio (WHR), body mass index (BMI), and eventual chest shape abnormalities, such as straight back syndrome (SBS) and pectus excavatum (PE), or inherited connective tissue disorder, such as Marfan Syndrome (MFS), asssociated with MVP]; (3) the prevalence of the most common cardiovascular risk factors (hypertension, smoking, type 2 diabetes mellitus, and dyslipidemia); (4) auscultatory findings (mid-systolic click and/or late systolic murmur) and hemodynamics (heart rate, systolic and diastolic blood pressure); (5) subjective symptoms, such as chest pain, palpitations, dyspnea, and/or syncope; (6) electrocardiographic (ECG) findings, particularly ST-T-wave abnormalities in inferior leads, ventricular premature beats (VPBs), atrial premature beats (APBs), and/or atrial fibrillation (AF); (7) left ventricular (LV) internal dimensions, LV systolic and diastolic function, and left atrial (LA) size assessed by 2D-TTE; (8) MVP prevalence in each study group; (9) complications associated with MVP, including arrhythmias, MR, IE, HF, and sudden cardiac death; (10) concomitant valvulopathies; (11) current medical treatment; and (12) follow-up data (if any). A third author (G.L.N.) checked the extracted data for accuracy and resolved possible discrepancies between reviewers.

### 2.4. Risk of Bias Assessment

Articles included in this systematic review were assessed for risk of bias (RoB) using the National Institutes of Health (NIH) Quality Assessment Tool for Observational Cohort and Cross-Sectional Studies [16]. All the studies were assigned a “yes”, “no”, or “other” to each of the 14 criteria outlined in the appraisal tool. Then, by considering each criterion, the investigators evaluated the overall quality of the study and assigned an overall “good” (met 11–14 criteria), “fair” (met 6–10 criteria), or “poor” (met 0–5 criteria) rating to each study. The quality rating was independently estimated by two authors (A.S. and G.L.N.). Any disagreement was resolved by consensus.

**Figure 1:**
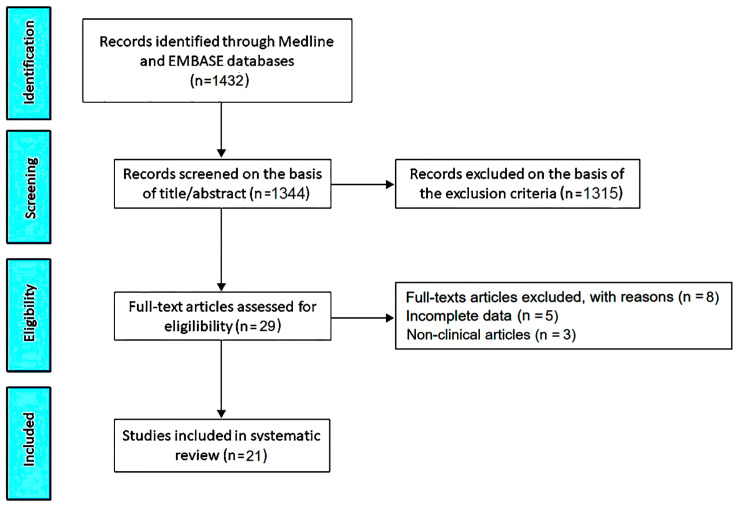
Flow diagram used for identifying the included studies.

The PRISMA flow diagram used to identify the included studies is depicted in Figure 1.

## 3. Results

The initial search yielded a total of 1432 studies. Of those, 88 (6.1%) were removed as duplicates. After screening titles and abstracts, a further 1315 studies (91.8%) were removed on the basis of the exclusion criteria. The evaluation of the full text of the remaining 29 studies (2%) resulted in eight additional exclusions (0.6%). A total of 21 studies (1.5%) [12,17,18,19,20,21,22,23,24,25,26,27,28,29,30,31,32,33,34,35,36] were thus included in this systematic review, totaling 1354 MVP individuals out of 63,723 participants.

The clinical characteristics and main findings of the included studies are summarized in Table 1.

The included studies were published between 1976 and 2021. Thirteen studies were performed in the USA, two in Italy, and one in Sweden, India, Greece, Canada, Turkey, and Taiwan.

The average population size was 3034 individuals (range of 100–24,265). Among the included studies, eight evaluated the MVP prevalence in population studies, such as the Framingham Heart Study [12,21], the Coronary Artery Risk Development in Young Adults (CARDIA) study [27], the Strong Heart Study [28], the Study of Health Assessment and Risk in Ethnic groups (SHARE study) [30], the Cardiorespiratory Fitness and Hospitalization Events in Armed Forces (CHIEF) study [35], and in large multicentric cohorts of ambulatory individuals [31,32]. The remaining thirteen studies analyzed monocentric cohorts of ambulatory patients, including a limited number of individuals.

The mean age of the individuals analyzed by the included studies was 37.4 yrs (range of 8–62.4 yrs). The average percentage of females was 53.1% (range of 0–100%).

The cohorts of individuals enrolled in the echocardiographic studies were examined by only M-mode echocardiography in six studies, both M-mode and 2D-echocardiography in seven studies, only 2D-echocardiography in eight studies, and both resting and exercise stress echocardiography (ESE) in one study. The selected individuals also underwent phonocardiogram in three studies, ECG in eight studies, chest X-rays (CXRs) in three studies, Holter ECG monitoring in two studies, and finally a treadmill exercise test in one study. No study used 3D-echocardiography.

Concerning the methodological assessment of MVP, the ten studies performed between 1976 and 1998 used poorly defined M-mode and 2D-echocardiographic diagnostic criteria, whereas the eleven studies conducted between 1999 and 2021 analyzed the MVP prevalence among heterogeneous cohorts of individuals by using more specific 2D-echocardiographic criteria, based on mitral annular nonplanarity (saddle-shaped configuration) derived from the studies highlighting the three-dimensional shape of the mitral annulus [9,10]. According to M-mode criteria, a late or pansystolic bowing of the mitral valve leaflets >2 mm below the C-D line was considered as diagnostic of MVP [5,6], whereas the older 2D-echocardiographic diagnostic criteria considered the displacement of the anterior leaflet in the apical four-chamber view as a sufficient condition for MVP diagnosis [7,8]. Conversely, the new 2D-echocardiographic criteria defined MVP as the systolic billowing of one or both of the mitral leaflets > 2 mm above the mitral annulus, always confirmed by examination of the parasternal long-axis view [12]. A maximal leaflet thickness of ≥5 mm identified classic or myxomatous MVP, whereas a leaflet thickness of <5 mm was diagnostic of non-classic MVP.

The overall pooled MVP prevalence among the included studies was 4.9% (range of 0.6–21%). When dividing the studies into two groups according to the echocardiographic criteria used for MVP diagnosis (less specific older or more specific new criteria, respectively), the estimated average MVP prevalence was 7.8% (range of 2–21%) for the older studies (performed between 1976 and 1999), which decreased to 2.2% (range of 0.6–4.2%) in the more recent studies (conducted between 1999 and 2021).

The higher prevalences of MVP were reported by the studies involving “self-referred” or “self-selected” healthy volunteers [17,18,22] and hospital-based studies, in which patients were commonly referred for complications under investigation [25]. In this regard, the average MVP prevalence was 63% among MVP individuals with ruptured mitral chordae tendineae [25], 55% among MVP individuals with moderate-to-severe MR [25], 35% among young adults aged 10 to 18 yrs [24], 21% among healthy females aged 17–35 yrs [17], 16.2% among children aged 9–12 yrs [26], and 16% among individuals affected by IE [25]. Moreover, the studies performed before the year 1999 described a higher MVP prevalence in females compared with male participants, with a peak in early adulthood and a subsequent decline [21].

On the contrary, the studies performed after the year 1999 detected a low prevalence of MVP in the general community, regardless of age, gender, and ethnicity. Notably, similar MVP prevalences were reported by those studies evaluating MVP prevalence in middle-aged individuals [31] or healthy teenagers [32], in males vs. females [12,31,32,35], in black vs. white individuals [27], or in different ethnicities [28,30]. Interestingly, Karavidas AI et al. [29] found a significantly higher prevalence of classic myxomatous MVP in males than in females. The MVP individuals described by the more recent studies were more likely to be affected by mild-to-moderate MR [12,27,28] with a low frequency of serious complications and a low probability of adverse outcomes over mid-to-long term follow-up periods [34,36].

All the included studies were primarily focused on the assessment of MVP prevalence among various cohorts of individuals, whereas information concerning anthropometrics, cardiovascular disease burden, auscultatory findings and hemodynamics, symptoms, ECG and echocardiographic findings, MVP complications, and concomitant valvulopathies were provided by a percentage of studies ranging from 9.5% and 38.1% of the total (Table 2).

MVP individuals were generally described as tall and lean, with a lower WHR and BMI in comparison to non-MVP individuals. Several chest shape abnormalities, particularly SBS, various degrees of anterior chest wall deformity ranging from concave-shaped chest wall conformation to severe forms of PE, and/or inherited connective tissue disorders, such as MFS, were described in approximately one-third of MVP subjects.

The cardiovascular disease burden was low due to the low prevalence of the most common cardiovascular risk factors, especially type 2 diabetes (average prevalence of 2.7%), and optimal blood pressure values detected in MVP participants. A coronary artery disease (CAD) history was detected in only 2.7% (range of 0–6.7%) of MVP individuals. The typical auscultatory findings associated with MVP were predominantly reported by the phonocardiographic studies performed between 1976 and 1998. According to these studies, mid-systolic click and/or late systolic murmur were detectable in 63.8% of MVP individuals (range of 33.7–80%). Conversely, the most recent studies conducted after 1999 made poor mention of the typical apical systolic murmur associated with MVP, a diagnostic test deemed by the authors considerably inferior to 2D-TTE.

Concerning symptoms, approximately one-third of MVP participants were symptomatic for palpitations, while dyspnea, nonspecific chest pain, and syncope were less frequently reported.

The analysis of ECGs revealed nonspecific ST-T-wave abnormalities in inferior leads in 28.1% (range of 0–73%) of MVP individuals and VPBs in 16.6% (range of 0–48.7%), whereas APBs were rarely detected.

On conventional TTE, compared to non-MVP individuals, those with MVP were generally diagnosed with similar LV size, similar LV wall thickness, and similar LV systolic and diastolic function. Interestingly, the two studies that measured also the antero-posterior (A-P) thoracic diameter, either invasively by CXR [17] or noninvasively by the modified Haller index (MHI) [36], found reduced LV internal dimensions among MVP individuals. Differently from these studies, Savage DD et al. [21] observed increased LV internal size in MVP individuals, particularly males.

MVP was found to be a benign condition. Indeed, it was commonly associated with a mild-to-moderate MR degree, described in 58.6% (range of 34.8–90%) of studies. MVP complications, such as severe MR, IE, HF, stroke, and AF, were rarely detected, affecting a percentage of MVP patients ranging from 0.4% to 4.7%.

Among the concomitant valvulopathies, MVP tended to be associated with tricuspid regurgitation secondary to tricuspid valve prolapse, in 41.4% (range of 7.1–75.6%) of patients.

With regards to the screening exercise tests for detecting myocardial ischemia in MVP patients, exercise stress testing revealed the frequent occurrence of VPBs during the recovery period with no evidence of ischemia-induced ST-segment changes [17], whereas ESE showed a high prevalence of negative ESE [36].

Information concerning follow-up data was scanty. Caselli S et al. [34] detected an increased prevalence of major adverse cardiovascular events (MACEs) among MVP athletes with moderate-to-severe MR, mitral annular disjunction (MAD), and ventricular arrhythmias (VAs) on twenty-four-hour Holter ECG monitoring, whereas our study group [36] demonstrated a low prevalence of MACEs among MVP individuals with a concave-shaped chest wall, as noninvasively assessed by a modified Haller index > 2.5 [37].

No cases of sudden cardiac death were detected among MVP individuals by the included studies.

Finally, only two studies provided information about the medical treatment of MVP individuals. On average, only one-fifth of MVP patients were treated with cardio-protective drugs, such as beta blockers, angiotensin-converting enzyme (ACE)-inhibitors, and calcium channel blockers, whereas diuretics were rarely prescribed.

Regarding the RoB assessment, the NIH quality rating was estimated as good for three studies and fair for eighteen studies (Table 3). The Cohen’s Kappa coefficient for the agreement between the reviewers in the RoB assessment was interpreted as a substantial agreement, k = 0.79.

## 4. Discussion

### 4.1. The Main Findings of the Present Systematic Review

This systematic review, which analyzed the main findings of 21 echocardiographic studies evaluating MVP prevalence in various cohorts of individuals throughout a 45-year period, highlighted a more than three-fold higher prevalence of MVP in the general community assessed by older studies (performed between 1976 and 1998) in comparison to that reported by more recent studies (conducted between 1999 and 2021). Based on the results of the older echocardiographic studies, MVP prevalence significantly increased among individuals with flail mitral valve, moderate-to-severe MR, and IE. In addition, MVP was frequently detected in healthy, young adult females and rarely among elderly individuals. The echocardiographic studies performed between 1999 and 2021 revealed a significantly lower MVP prevalence in various series of individuals, including large population cohorts. MVP prevalence was similar among the included studies, independent of age, gender, and ethnicity. In recent years, the typical auscultatory findings associated with MVP have lost clinical relevance due to the reliability and high diagnostic performance of 2D-TTE; indeed, MVP may be detected on 2D-TTE and also in the presence of a normal physical examination. Compared to non-MVP individuals, those with MVP have a number of anthropometric, clinical, ECG, and echocardiographic characteristics that may be considered pathognomonic of this valvular disorder. Notably, they are generally tall and thin, with a low prevalence of type 2 diabetes and CAD, paucisymptomatic for palpitations, dyspnea, and nonspecific chest pain, with nonspecific ST-T-wave abnormalities in inferior leads and isolated VPBs on resting ECG, with reduced LV internal size, preserved LV systolic function, and mild-to-moderate MR on 2D-TTE. Overall, MVP has a benign clinical course, with a low prevalence of relevant complications, such as severe MR, IE, HF, stroke, and AF, and a good prognosis over a mid-to-long-term follow-up period.

### 4.2. Possible Explanations for the Divergent Findings between the Studies Performed before and after the Year 1999

The true prevalence of MVP in the general population may have been systematically overestimated by the older echocardiographic studies performed before the year 1999 due to a number of technical issues. First, these studies were subject to selection bias in the cohorts of participants evaluated. Indeed, the studies that reported higher prevalences of this disorder included volunteers who were concerned about their cardiac status because of affected family members, who had a history of heart murmur or subtle symptoms, and who were well known to the examining physician [17,18,20]. Moreover, the high frequency of complications associated with MVP, such as severe MR, IE, and stroke, was generally described in patients selected from large clinics or hospital practices rather than from the general population [25]. Therefore, the high prevalence of MVP in such studies is likely not reflective of the real prevalence of this disorder in the general community.

Another major reason for the overestimation of the true prevalence of MVP in older studies can be attributed to the use of poorly defined M-mode and 2D-echocardiographic diagnostic criteria. With regard to M-mode echocardiography, several false positive diagnoses of MVP could be related to the inherent spatial limitations of this technique in representing the mitral valve leaflets, mitral annulus, and left atrium simultaneously; indeed, several artifacts may occur in M-mode echocardiography when the transducer is not perpendicular to the valve leaflets but has a high or low position relative to the mitral valve [38]. Therefore, the results of M-mode echocardiography vary widely depending on the orientation of the transducer [39]. Moreover, an inconsistent correlation of the M-mode echocardiographic pattern with auscultatory findings or clinical symptoms of MVP has been repeatedly reported [12,24,26].

**Figure 2:**
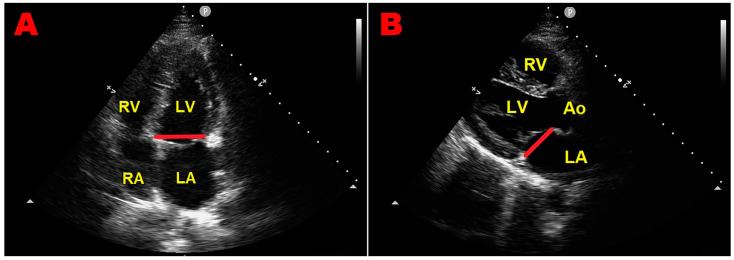
An example of superior anterior mitral leaflet displacement in the apical four-chamber view (panel (**A**)), absent in the parasternal long-axis view (panel (**B**)), accepted as the diagnostic standard of mitral valve prolapse before the year 1999. The red line indicates the mitral annular plane in each of the two orthogonal views. Ao, aorta; LA, left atrium; LV, left ventricle; RA, right atrium; RV, right ventricle.

The older 2D-echocardiographic studies evaluating MVP prevalence did not consider the saddle-like shape of the mitral annulus and used nonspecific 2D-echocardiographic criteria, including displacement of the anterior leaflet in the apical four-chamber view, to diagnose MVP. Studies describing the three-dimensional shape of the mitral annulus [9,10] have allowed for the refining of the 2D-echocardiographic criteria. Based on the saddle-shaped configuration of the mitral annulus, leaflets can appear to ascend above the mitral annulus in the apical four-chamber view without actual leaflet displacement above the high points of the annulus contained in the long-axis view [9,10,13] (Figure 2).

**Figure 3:**
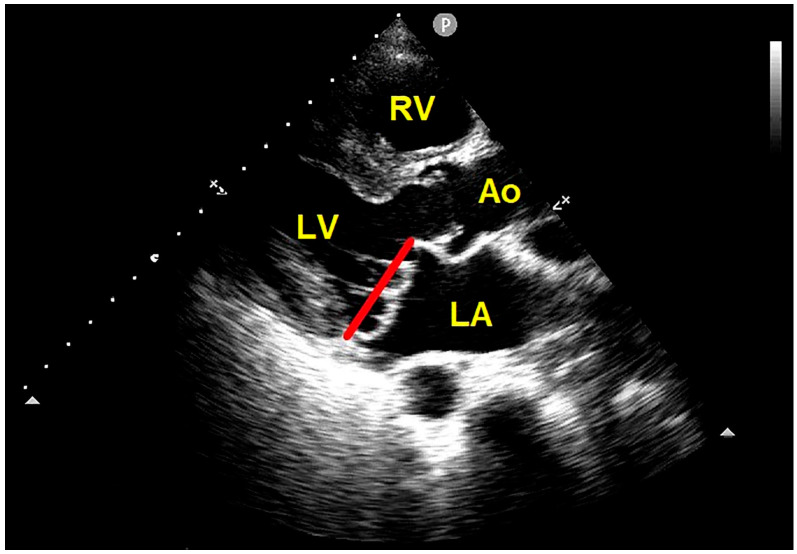
Transthoracic echocardiography. Parasternal long-axis view showing the systolic billowing of both mitral leaflets > 2 mm above the mitral annulus plane, compatible with bileaflet MVP. The red line indicates the plane of the mitral annulus. Ao, aorta; LA, left atrium; LV, left ventricle; MVP, mitral valve prolapse; RV, right ventricle.

Eliminating the use of the apical four-chamber view reduces false positive diagnoses [13]. According to the more specific 2D-echocardiographic criteria, MVP is actually defined as the systolic displacement of one or both mitral leaflets > 2 mm above the mitral annulus plane, visualized from the parasternal long-axis view [12] (Figure 3).

The increased prevalence of MVP among healthy young adult females reported by the older studies was likely related to both selection bias and the use of poorly defined M-mode and/or 2D-echocardiographic criteria for MVP adopted by these studies. Similarly, the high frequency of complications associated with MVP described before the year 1999 [1,2,3,4] could be ascribed to the improper selection of cohorts of individuals referred to large hospital centers for complications under investigation.

### 4.3. Implications for Clinical Practice

**Figure 4:**
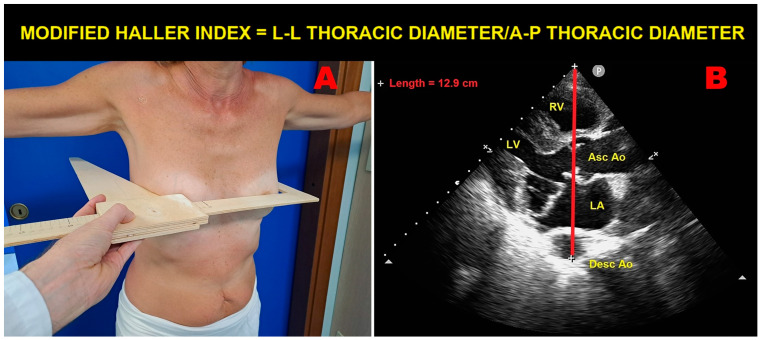
An example of modified Haller index assessment in an individual with mitral valve prolapse. Panel (**A**): The L-L thoracic diameter, measured with the individual in the standing position and with open arms, using a rigid ruler in centimeters coupled to a level (the measuring device) placed at the distal third of the sternum at the point of maximum depression of the sternum. Panel (**B**): The A-P thoracic diameter, obtained with the individual in the left-lateral decubitus position during conventional transthoracic echocardiography by placing a 2.5 mHz transducer near the sternum in the left third or fourth intercostal space to obtain a parasternal long-axis view, and measuring the distance between the true apex of the sector and the anterior surface of the vertebral body. The vertebral body is identified by using, as a reference point, the posterior wall of the descending thoracic aorta, visualized behind the left atrium. Ao, aorta; A-P, antero-posterior; Asc, ascending; Desc, descending; LA; left atrium; L-L, latero-lateral; LV, left ventricle; RV, right ventricle.

The echocardiographic detection of MVP should be specifically focused on individuals who are tall and lean, with a low BMI and WHR, and with various degrees of anterior chest wall deformity. In this regard, it is noteworthy that MVP is commonly associated with several thoracic skeletal abnormalities (TSAs), such as PE, pectus carinatum, scoliosis, SBS, and connective tissue disorders, such as MFS [40]. These TSAs may be encountered in clinical practice more frequently than commonly expected, particularly in their forms of minor severity. The objective evidence of a concave-shaped chest wall in an individual who is tall and thin may indicate the clinical suspicion of MVP, especially in the presence of frequent isolated VPBs and nonspecific ST-T-wave abnormalities in inferior leads on resting ECGs. The MHI represents a simple and noninvasive method to quantify the degree of anterior chest wall deformity. It is obtained by dividing the latero-lateral maximum external thoracic diameter by the A-P minor internal thoracic diameter, without using CXR, thus avoiding exposure to ionizing radiation (Figure 4).

MVP individuals are generally diagnosed with an MHI > 2.5 due to a narrow A-P thoracic diameter (generally <13.5 cm) [41]. Both MVP and anterior chest wall deformity may have a common developmental origin, since the primordia of the mitral valve undergo differentiation to their final form around the 5th to 6th week of gestation, the same period during which the vertebral column and the thoracic cage start their chondrification and ossification. A defect in growth patterns at this stage of development might affect both the mitral valve and the bony thorax [42,43]. The narrow A-P thoracic diameter may explain the reduced LV internal dimensions detected in several MVP individuals and, most of all, the frequent occurrence of arrhythmias detected in such individuals. Indeed, the basal sternal compression continuously exerted on cardiac chambers may have a preponderant role in generating mitral annular distortion and MVP, with consequent abnormal traction on papillary muscle tips and increased occurrence of both VPBs and symptoms, such as palpitations, atypical chest pain, and dyspnea. In addition, it is not uncommon to detect wall motion abnormalities in MVP individuals on both resting 2D-TTE and ESE. A careful evaluation of these myocardial asynergies may allow them to be considered more properly as areas of intra- and/or inter-ventricular dyssynchrony due to modified heart motion, cardiac rotation, and tilting, rather than areas of hypokinesia or akinesia due to myocardial ischemia. The dyssynchronous cardiac motion within a narrow A-P thoracic diameter may be enhanced by physical exercise, in the absence of obstructive CAD [44,45]. This mechanical theory is supported by the evidence of a weak association between MVP and CAD [12,46,47].

The studies included in this systematic review have also highlighted that the MR associated with MVP is generally mild or mild-to-moderate, and is rarely severe. MR severity may be overestimated by 2D-TTE, particularly in MVP individuals with a concave-shaped chest wall, due to the small size of all cardiac chambers, mostly the left atrium as the receiving chamber [48]. In MVP individuals, jets are often very eccentric, and poor alignment with an eccentric jet may lead to overestimation of both the vena contract and the effective regurgitant orifice area, especially when the left atrial size is small [49,50].

From a clinical viewpoint, in the presence of nonspecific ST-T-wave abnormalities on ECG, atypical chest pain, and mild-to-moderate MR due to MVP on 2D-TTE, ESE could be particularly useful for excluding obstructive CAD and for evaluating the impact of MR on pulmonary hemodynamics. As detected by our study group in a large cohort of individuals referred to perform ESE for suspected CAD [51], a preliminary chest shape assessment by the MHI method together with pre-test probability estimation might help clinicians to identify, among MVP individuals, those with a lower probability of exercise-induced myocardial ischemia and with an increased probability of non-hemodynamically significant MR.

### 4.4. Syndromic Mitral Valve Prolapse

In the majority of cases, MVP is isolated and not associated with other conditions. However, MVP can also be seen as part of connective tissue disorders, such as Marfan syndrome (MFS), Loeys–Dietz syndrome (LDS), Ehlers–Danlos syndrome (EDS), osteogenesis imperfecta (OI), and aneurysms–osteoarthritis syndrome [52]. The prevalence of MVP is much higher in these conditions compared to the general population [53]. Notably, MVP may affect 40% of MFS patients [54], 15–41% of LDS patients [55], 6% of EDS patients [56], 7% of OI patients [57], and 45% of patients affected by aneurysms–osteoarthritis syndrome [58]. MFS, LDS, and EDS patients have an increased risk of aortic dissection and severe MR due to the progressive degeneration of the valve and chordae, with myxomatous infiltration and fibroelastic and collagen alterations [59]. Moreover, the association between MAD, arrhythmic events, and mitral valve surgery has been consistently reported in the MFS population [60,61]. MVP has also been reported in 2% of patients with long QT syndrome (LQTS) [62] and in 9–26% of patients with QT interval prolongation [63]. Finally, MVP has been associated with left ventricular noncompaction (LVNC) and ion channelopathies [64]. Two authors have described the concomitance of sinus bradycardia, LVNC, and MVP [65,66]. This overlapping phenotype is caused by the mutations and dysfunction of the hyperpolarization-activated cyclic nucleotide channel 4 (HCN4), a major constituent of the pacemaker current (If) in the sinoatrial node [67]. QT interval prolongation and cardiac ion channelopathies may increase the risk of malignant VAs and sudden cardiac death (SCD) in MVP individuals [68].

### 4.5. Mitral Valve Prolapse Complications

Even if MVP is generally a benign condition, a number of serious complications may occur in individuals with MVP.

MVP individuals have an annual risk of SCD, ranging from 0.2% to 1.9% [63,69]. A subset of patients with MVP and an increased incidence of malignant VAs, and SCD has recently been identified [70]. These individuals have myxomatous and bileaflet MVP without significant MR and with the presence of MAD [63]. The latter may trigger frequent and/or complex VAs due to the abnormal tugging on the submitral apparatus resulting in papillary muscle hypertrophy and fibrosis [71,72]. The wider the magnitude of MAD, the higher the incidence of VAs. MVP individuals with myxomatous and bileaflet MVP and with MAD presence should undergo regular 24 h ECG Holter monitoring at diagnosis and during follow-up, with a detailed evaluation of arrhythmias [73]. In selected cases, particularly in MVP patients with abnormal electrocardiographic or Holter findings, a multimodality imaging approach comprehensive of cardiac magnetic resonance (CMR) would improve MAD detection and better define its circumferential extent [74].

The clinical course of MVP may be complicated by the occurrence of AF, particularly in those patients with severe MR [75,76]. AF is a negative prognostic factor, given that patients with AF at the time of MR surgery have worse outcomes compared with patients without AF [77].

MVP may also cause LV dysfunction, and systolic heart failure [78]. The chronic volume overload increases LV wall stress, LV hypertrophy, fibrosis, and LV afterload, with a gradually worsening systolic function [79]. In addition, MR secondary to MVP may determine progressive LA pressure and volume overload [80], resulting in LA enlargement and contributing to AF occurrence [81].

MR is a natural consequence of MVP [82] and MVP is the most common cause of severe MR requiring surgery [83,84]. Worsening regurgitation causes an increase in volume overload, which further increases the annulus diameter and further worsens the amount of regurgitation [85]. MR due to MVP may also develop abruptly as a result of papillary muscle, or chordal rupture (“flail mitral valve”). In this case, MR develops acutely without appropriate compensation by the LA. This leads to acute LA volume overload and causes pulmonary edema and symptoms of respiratory distress [86]. Mitral valve surgery is highly effective at preventing mortality in patients with severe MR [87]. Early surgery is recommended in patients with MR secondary to flail leaflets and is generally associated with a good outcome [88].

Finally, MVP patients have a 3- to 8-fold increased risk of developing IE, although the estimated annual risk is still quite low, at 0.02% [89]. The main risk factors for EI in MVP patients are MR presence, advanced age, male sex, and leaflet thickening/redundancy [89]. Based on the new 2D-echocardiographic criteria for MVP diagnosis, since 2008 [90], prophylaxis against IE has no longer been recommended for patients with MVP without MR or without thickened leaflets on TTE. Conversely, MVP patients with thickened leaflets and documented MR on TTE may benefit from antibiotics during procedures that often lead to bacteremia.

The medical treatment of hemodynamically relevant MR due to MVP is based on angiotensin-converting enzyme inhibitors which promote a reduction in cardiac after-load by decreasing systemic vascular resistance, beta blockers which promote negative inotropic and chronotropic effects, decreasing cardiac demand, and oxygen demand and loop diuretics which promote a reduction in cardiac preload by decreasing the total blood volume [91].

### 4.6. Limitations of the Included Studies

The present systematic review analyzed a heterogeneous group of echocardiographic studies conducted over a 45-year period. The studies conducted before the year 1999 were limited by potential selection bias, hospital- or referral-based series, the use of several techniques (phonocardiographic, auscultatory and M-mode, and/or 2D-echocardiographic examinations) for evaluating MVP prevalence, and finally by the use of less specific echocardiographic criteria for MVP diagnosis. On the other hand, the more recent studies (performed after the year 1999) analyzed large population cohorts by using more specific and standardized 2D-echocardiographic criteria, thus providing a univocal and more reliable estimation of the true MVP prevalence in the general community.

To date, as far as we know, no previous study has evaluated MVP prevalence in the general population by using 3D-echocardiography. By providing the “en-face” view of the valve from the atrial perspective (the so-called “surgical view”), 3D-echocardiography allows a comprehensive evaluation of the mitral valve morphology and a simultaneous visualization of all mitral scallops. Both MVP and its complications may be diagnosed with high specificity by 3D-echocardiography [92].

## 5. Conclusions

In the general population, MVP is an uncommon echocardiographic finding and the low prevalence of this disorder is similar in different age, gender, and ethnic groups.

MVP is generally a benign condition, commonly associated with a narrow A-P thoracic diameter, isolated VPBs, mild-to-moderate MR, the reduced burden of cardiovascular disease, and a low frequency of complications, such as severe MR, IE, HF, and AF.

MVP individuals with myxomatous and bileaflet MVP without significant MR and with MAD presence and those affected by syndromic MVP have an increased risk of malignant VAs and SCD and need closer clinical follow-up.

The innovative MHI method could be useful for identifying, among MVP individuals, those with a concave-shaped chest wall (MHI > 2.5 and/or A-P thoracic diameter < 13.5 cm) who have an increased probability of being diagnosed with a benign phenotype of MVP, characterized by nonspecific ST-T-wave abnormalities on ECG, areas of intra- and/or inter-ventricular dyssynchrony on 2D-TTE, and good outcome over mid-to-long term follow-up periods.

## Figures and Tables

**Table 1:** The summary and main findings of the included studies. Abbreviations: 2D-echo, two-dimensional echocardiography; CXR, chest X-rays; ECG, electrocardiogram; ESE, exercise stress echocardiography; IE, infective endocarditis; M-mode echo, M-mode echocardiography; MR, mitral regurgitation; MVP, mitral valve prolapse; N and S, North and South.

Study Name, Publication Year, and Country	Population Size (% Females) and Subgroups	Mean Age (yrs)	Screening Methods	MVP Individuals (MVP Rate %)
Markiewicz W et al. (1976), USA [17]	100 (100)	23.5	Phonocardiogram, ECG, M-mode echo, exercise stress test, CXR	21 (21)
Procacci PM et al. (1976), USA [18]	1169 (100)	32	ECG, M-mode echo, CXR	74 (6.3)
McLarin C et al. (1979), USA [19]	1823 (0)	41	M-mode echo	56 (3)
Wann LS et al. (1983), USA [20]	100 (100)	25	Phonocardiogram, ECG, M-mode echo, 2D-echo	2 (2)
Savage DD et al. (1983), USA [21]	4967 (55.2) 2036 elderly 2931 offspring	57 70 elderly 44 offspring	M-mode echo	264 (5) 56 (3) elderly 208 (7) offspring
Bryhn M et al. (1984), Sweden [22]	201 (49.7) 101 males 100 females	25 19.5 males 30.5 females	M-mode echo	13 (6.5) 5 (6.9) males 8 (8) females
Lee WR et al. (1985), USA [23]	200 (50) 100 males 100 females	44	Phonocardiogram, ECG, M-mode echo, CXR	5 (2.5) 2 (2) males 3 (3) females
Warth DC et al. (1985), USA [24]	193 (49.2) 78 (0–2 yrs) 65 (2–6 yrs) 21 (6–10 yrs) 29 (10–18 yrs)	4.5	2D-echo	25 (13) 1 (1.3) (0–2 yrs) 9 (14) (2–6 yrs) 5 (24) (6–10 yrs) 10 (35) (10–18 yrs)
Devereux RB et al. (1986), USA [25]	2483 (56.9) 67 IE 31 MR 43 Flail 2342 Ctrls	47 38 IE 50 MR 56 Flail 54 Ctrls	M-mode echo, 2D-echo	147 (5.9) 11 (16) IE 17 (55) MR 27 (63) Flail 92 (4) Ctrls
Gupta R et al. (1992), India [26]	213 (40.4) 96 (3–5.9 yrs) 80 (6–8.9 yrs) 37 (9–12 yrs)	8	M-mode echo, 2D-echo	28 (13.1) 13 (13.5) (3–5.9 yrs) 9 (11.2) (6–8.9 yrs) 6 (16.2) (9–12 yrs)
Freed LA et al. (1999), USA [12]	3491 (52.8) 1646 males 1845 females	54.7	ECG, 2D-echo	84 (2.4) 34 (2.1) males 50 (2.7) females
Flack JM et al. (1999), USA [27]	4136 (55) 1996 black 2140 white	29.7	M-mode echo, 2D-echo	26 (0.6) 10 (0.5) black 16 (0.7) white
Devereux RB et al. (2001), USA [28]	3340 (62.2) 1140 Oklahoma 1137 Arizona 1063 N and S Dakota	60	M-mode echo, 2D-echo	57 (1.7) 31 (2.6) Oklahoma 7 (0.6) Arizona 18 (1.6) N and S Dakota
Karavidas AI et al. (2002), Greece [29]	2080 (63.1)	51.5	2D-echo	41 (1.97) 22 (1.07) classic MVP 19 (0.9) non-classic MVP
Theal M et al. (2004), Canada [30]	972 (48) 336 South Asian 322 European 314 Chinese	49	ECG, M-mode echo, 2D-echo	26 (2.7) 9 (2.7) South Asian 10 (3.1) European 7 (2.2) Chinese
Hepner AD et al. (2007), USA [31]	24265 (53) 11.339 males 12.926 females	49.6	M-mode echo, 2D-echo	135 (0.6) 80 (0.7) males 55 (0.4) females
Sattur S et al. (2010), USA [32]	2072 (33) 1382 males 690 females	16.1	2D-echo	14 (0.7) 5 (0.4) males 9 (1.3) females
Güvenç TS et al. (2012), Turkey [33]	936 (60) 442 sea level 494 high altitude	50.5 50 sea level 51 high altitude	M-mode echo, 2D-echo	39 (4.2) 9 (2) sea level 30 (6.1) high altitude
Caselli S et al. (2018), Italy [34]	7449 (33)	30	ECG, 2D-echo, Holter ECG	215 (2.9)
Liu PY et al. (2021), Taiwan [35]	2442 (11.8) 2154 males 288 females	26.5	2D-echo	82 (3.3) 77 (3.6) males 5 (1.7) females
Sonaglioni A et al. (2021), Italy [36]	1091 (42.8)	62.4	ECG, 2D-echo, ESE	35 (3.2)

**Table 2:** The clinical and instrumental characteristics of the MVP individuals analyzed by the included studies. Data are expressed as the median andinterquartile range. ACE, angiotensin-converting enzyme; AF, atrial fibrillation; APBs, atrial premature beats; BMI, body mass index; CAD, coronary artery disease; DBP, diastolic blood pressure; ECG, electrocardiogram; HF, heart failure; HR, heart rate; IE, infective endocarditis; MR, mitral regurgitation; MVP, mitral valve prolapse; SBP, systolic blood pressure; TR, tricuspid regurgitation; VPBs, ventricular premature beats; WHR, waist-to-hip ratio.

	MVP Individuals	Number of Studies for Parameters Assessed (%)
**Demographics**		
Females (%)	53.1 (0–100)	21 (100)
Mean age (yrs)	37.4 (8–62.4)	21 (100)
**Anthropometrics and chest shape conformation**		
WHR	0.87 (0.77–0.94)	3 (14.3)
BMI (Kg/m^2^)	24.8 (22–28)	6 (28.6)
Chest shape abnormalities (%)	26 (0–100)	6 (28.6)
**Cardiovascular risk factors and cardiovascular disease burden**		
Hypertension (%)	17.2 (0–32)	5 (23.8)
Smoking (%)	20.3 (13.9–28)	4 (19.0)
Type 2 diabetes (%)	2.7 (0.7–4.8)	4 (19.0)
Dyslipidemia (%)	25.1 (21.6–28.6)	2 (9.5)
CAD (%)	2.7 (0–6.7)	6 (28.6)
**Auscultatory findings and hemodynamics**		
Mid-systolic click and/or late systolic murmur (%)	63.8 (33.7–80)	7 (33.3)
HR (bpm)	72.7 (65–87.8)	4 (19.0)
SBP (mmHg)	116.9 (102–127)	8 (38.1)
DBP (mmHg)	72.9 (68–81)	8 (38.1)
**Symptoms**		
Nonspecific chest pain (%)	11.9 (1.3–26.8)	6 (28.6)
Palpitations (%)	28.2 (20–35.8)	4 (19.0)
Dyspnea (%)	14.4 (9–22.5)	3 (14.3)
Syncope (%)	10.1 (0–26.8)	3 (14.3)
No symptoms (%)	21.3 (18–24.6)	2 (9.5)
**ECG findings**		
ST-T-wave abnormality in inferior leads (%)	28.1 (0–73)	6 (28.6)
VPBs (%)	16.6 (0–48.7)	6 (28.6)
APBs (%)	3.2 (0–10.1)	5 (23.8)
**MVP prevalence**		
Overall MVP prevalence (%)	4.9 (0.6–21)	21 (100)
MVP prevalence before the year 1999 (%)	7.8 (2–21)	10 (47.6)
MVP prevalence after the year 1999 (%)	2.2 (0.6–4.2)	11 (52.4)
**MVP complications**		
Mild-to-moderate MR (%)	58.6 (34.8–90)	6 (28.6)
Severe MR (%)	4.7 (0–9)	5 (23.8)
IE (%)	3.8 (0–16)	5 (23.8)
HF (%)	0.7 (0–2)	3 (14.3)
Stroke (%)	0.4 (0–1.2)	3 (14.3)
AF (%)	0.6 (0–1.2)	2 (9.5)
**Concomitant valvulopathies**		
TR (%)	41.4 (7.1–75.6)	2 (9.5)
**Current medical treatment**		
Beta blockers (%)	16.9 (4–29.9)	2 (9.5)
ACE-inhibitors (%)	16.1 (5–27.2)	2 (9.5)
Diuretics (%)	4.3 (3.6–5)	2 (9.5)
Calcium channel blockers (%)	17.1 (16.2–18)	2 (9.5)

**Table 3:** Quality assessment of the included studies. Q1: Was the research question or objective in this paper clearly stated? Q2: Was the study population clearly specified and defined? Q3: Was the participation rate of eligible persons at least 50%? Q4: Were all the subjects selected or recruited from the same or similar populations (including the same time period)? Were inclusion and exclusion criteria for being in the study prespecified and applied uniformly to all participants? Q5: Was a sample size justification, power description, or variance and effect estimates provided? Q6: For the analyses in this paper, was the exposure(s) of interest measured prior to the outcome(s) being measured? Q7: Was the timeframe sufficient so that one could reasonably expect to see an association between exposure and outcome if it existed? Q8: For exposures that can vary in amount or level, did the study examine different levels of the exposure as related to the outcome (e.g., categories of exposure, or exposure measured as a continuous variable)? Q9: Were the exposure measures (independent variables) clearly defined, valid, reliable, and implemented consistently across all study participants? Q10: Was the exposure(s) assessed more than once over time? Q11: Were the outcome measures (dependent variables) clearly defined, valid, reliable, and implemented consistently across all study participants? Q12: Were the outcome assessors blinded to the exposure status of participants? Q13: Was the loss to follow-up after baseline 20% or less? Q14: Were key potential confounding variables measured and adjusted statistically for their impact on the relationship between exposure(s) and outcome(s)? Good: met 11–14 criteria, fair: met 6–10 criteria, poor: met 0–5 criteria. NA, not applicable; NIH = National Institutes of Health; NS, not specified.

NIH Quality Assessment Tool for Observational Cohort and Cross-Sectional Studies															
Study Name	Q 1	Q 2	Q 3	Q 4	Q 5	Q 6	Q 7	Q 8	Q 9	Q 10	Q 11	Q 12	Q 13	Q 14	Quality
Markiewicz W et al. (1976) [17]	Yes	Yes	Yes	Yes	NA	Yes	Yes	No	Yes	NA	Yes	Yes	NS	No	9 (Fair)
Procacci PM et al. (1976) [18]	Yes	Yes	Yes	Yes	NA	Yes	Yes	No	Yes	NA	Yes	NS	NS	No	8 (Fair)
McLarin C et al. (1979) [19]	Yes	Yes	Yes	Yes	NA	Yes	Yes	No	Yes	NA	Yes	NS	NS	No	8 (Fair)
Wann LS et al. (1983) [20]	Yes	Yes	Yes	Yes	NA	Yes	Yes	No	Yes	NA	Yes	Yes	NS	No	9 (Fair)
Savage DD et al. (1983) [21]	Yes	Yes	Yes	Yes	NA	Yes	Yes	No	Yes	NA	Yes	NS	NS	Yes	9 (Fair)
Bryhn M et al. (1984) [22]	Yes	Yes	Yes	Yes	NA	Yes	Yes	No	Yes	NA	Yes	NS	NS	No	8 (Fair)
Lee WR et al. (1985) [23]	Yes	Yes	Yes	Yes	NA	Yes	Yes	No	Yes	NA	Yes	Yes	NS	No	9 (Fair)
Warth DC et al. (1985) [24]	Yes	Yes	Yes	Yes	NA	Yes	Yes	No	Yes	NA	Yes	NS	NS	No	8 (Fair)
Devereux RB et al. (1986) [25]	Yes	Yes	Yes	Yes	NA	Yes	Yes	Yes	Yes	NA	Yes	NS	Yes	Yes	11 (Good)
Gupta R et al. (1992) [26]	Yes	Yes	Yes	Yes	NA	Yes	Yes	No	Yes	NA	Yes	NS	NS	No	8 (Fair)
Freed LA et al. (1999) [12]	Yes	Yes	Yes	Yes	NA	Yes	Yes	Yes	Yes	NA	Yes	NS	Yes	Yes	11 (Good)
Flack JM et al. (1999) [27]	Yes	Yes	Yes	Yes	NA	Yes	Yes	No	Yes	NA	Yes	NS	Yes	Yes	10 (Fair)
Devereux RB et al. (2001) [28]	Yes	Yes	Yes	Yes	NA	Yes	Yes	Yes	Yes	NA	Yes	NS	Yes	Yes	11 (Good)
Karavidas AI et al. (2002) [29]	Yes	Yes	Yes	Yes	NA	Yes	Yes	No	Yes	NA	Yes	NS	NS	No	8 (Fair)
Theal M et al. (2004) [30]	Yes	Yes	Yes	Yes	NA	Yes	Yes	No	Yes	NA	Yes	Yes	NS	No	9 (Fair)
Hepner AD et al. (2007) [31]	Yes	Yes	Yes	Yes	NA	Yes	Yes	No	Yes	NA	Yes	NS	NS	No	8 (Fair)
Sattur S et al. (2010) [32]	Yes	Yes	Yes	Yes	NA	Yes	Yes	No	Yes	NA	Yes	NS	NS	No	8 (Fair)
Güvenç TS et al. (2012) [33]	Yes	Yes	Yes	Yes	NA	Yes	Yes	Yes	Yes	NA	Yes	NS	NS	No	9 (Fair)
Caselli S et al. (2018) [34]	Yes	Yes	Yes	Yes	NA	Yes	Yes	Yes	Yes	NA	Yes	NS	Yes	No	10 (Fair)
Liu PY et al. (2021) [35]	Yes	Yes	Yes	Yes	NA	Yes	Yes	Yes	Yes	NA	Yes	NS	NS	Yes	10 (Fair)
Sonaglioni A et al. (2021) [36]	Yes	Yes	Yes	Yes	NA	Yes	Yes	Yes	Yes	NA	Yes	NS	Yes	No	10 (Fair)

## Data Availability

Data extracted from included studies will be publicly available on Zenodo (https://zenodo.org), pending acceptance by the journal.
